# Facial feedback and autonomic responsiveness reflect impaired emotional processing in Parkinson’s Disease

**DOI:** 10.1038/srep31453

**Published:** 2016-08-11

**Authors:** Michela Balconi, Francesca Pala, Rosa Manenti, Michela Brambilla, Chiara Cobelli, Sandra Rosini, Alberto Benussi, Alessandro Padovani, Barbara Borroni, Maria Cotelli

**Affiliations:** 1 Research Unit in Affective and Social Neuroscience, Department of Psychology, Catholic University, Milan, Italy; 2 Neuropsychology Unit, IRCCS Centro San Giovanni di Dio Fatebenefratelli, Brescia, Italy; 3 Centre for Aging Brain and Neurodegenerative Disorders, Neurology unit, University of Brescia, Brescia, Italy

## Abstract

Emotional deficits are part of the non-motor features of Parkinson’s disease but few attention has been paid to specific aspects such as subjective emotional experience and autonomic responses. This study aimed to investigate the mechanisms of emotional recognition in Parkinson’s Disease (PD) using the following levels: explicit evaluation of emotions (Self-Assessment Manikin) and implicit reactivity (Skin Conductance Response; electromyographic measure of facial feedback of the zygomaticus and corrugator muscles). 20 PD Patients and 34 healthy controls were required to observe and evaluate affective pictures during physiological parameters recording. In PD, the appraisal process on both valence and arousal features of emotional cues were preserved, but we found significant impairment in autonomic responses. Specifically, in comparison to healthy controls, PD patients revealed lower Skin Conductance Response values to negative and high arousing emotional stimuli. In addition, the electromyographic measures showed defective responses exclusively limited to negative and high arousing emotional category: PD did not show increasing of corrugator activity in response to negative emotions as happened in heathy controls. PD subjects inadequately respond to the emotional categories which were considered more “salient”: they had preserved appraisal process, but impaired automatic ability to distinguish between different emotional contexts.

Parkinson’s Disease (PD) is the second most prevalent neurodegenerative disease and is characterized by the presence of rest tremor, rigidity, bradykinesia, gait impairment, and postural instability. Cognitive impairment, emotional disturbances and mood disorders often occur over disease course^1^,^2^,^3^,^4^,^5^. An important question is related to the presence of an effective impairment in facial expression and facial responsiveness in PD, clearly distinct from the basic motor deficits^6^,^7^. One of the research field that has not yet been fully explored is the emotional processing in PD, as it is not yet established whether the patients have lost the ability to feel the emotions *per se* or to decipher emotions in others (for a review see ref. 8). Previous studies mainly explored self-reported experiences (explicit measures), arguing for a general decreased response to negative and aversive stimuli. However, these researches did not consider the explicit emotional appraisal, i.e. the knowledge of the emotional meaning of the presented stimuli, that is mainly defined by valence and arousal^9^,^10^. In addition to the explicit appraisal, emotional experience might be further characterized by considering the implicit emotional reactivity in either unpleasant or pleasant contexts. In particular, electrodermal activity (Skin Conductance Response, SCR), is consistently modified by emotional valence and arousal, generally with greater responses (increased SCR) elicited in highly arousing contexts^11^,^12^. In PD, lower levels of physiological arousal compared with control subjects have been reported^13^,^14^. Indeed, no emotion-specific impairment was demonstrated but, in contrast, a general hypo-responsiveness to high-arousal negative patterns has been shown^15^. It has been supposed that this autonomic reduced activity could be specifically related to negative valence-stimuli, with a general decreasing of responses to negative and aversive stimuli due to the difficulty to link their self-evaluated emotional condition with their arousal-based physiological modifications.

Additionally, only few studies have investigated the role of electromyographic (EMG) facial feedback effect to determinate the impaired emotional behaviour in PD^16^,^17^. Facial feedback model has supposed that the facial response by some of the main facial muscles (such as the zygomaticus and corrugator muscles) may affect both the emotional appraisal and the physiological modulation^18^: this facial muscles activity would determine the “facial feedback” of emotions. Specifically, it was shown that the “facial feedback” of positive emotions is mainly supported by an increased zygomaticus muscle activity, whereas negative emotions are supported by increased corrugator muscle intensity^19^,^20^. Amimia is a typical facial trait of PD, due to akinesia and rigidity of facial muscles^21^,^22^. In line with “facial simulation” this muscles unresponsiveness may partially explain the central/peripheral emotional deficits for any emotional category, due to the fact that PD patients might show a reduced ability to mimic emotions by face^6^,^23^.

The aim of the present study was to comprehensively assess the mechanisms of emotional recognition in PD.

Twenty PD patients and 34 matched healthy controls (HC) were required to observe and evaluate affective pictures (high and low arousal positive images, high and low arousal negative stimuli and neutral stimuli) during physiological parameters recording. We assessed self-reported emotional recognition of valence and arousal (explicit appraisal), autonomic recordings of SCR and electromyographic recordings of facial feedback of the zygomaticus and corrugator muscles. Both the data resulting from the explicit evaluation of emotions (Self-Assessment Manikin - SAM) and the implicit reactivity (SCR and EMG) have been analyzed comparing the different emotion categories (high and low arousal positive images, high and low arousal negative stimuli and neutral stimuli) and the two experimental groups (PD patients vs. healthy controls).

Specifically, we aimed to investigate whether the blunted facial expressiveness may be emotional-specific and not solely related to motor deficits. According to this hypothesis, we would expect a specific reduced responsiveness in facial muscles, such as the corrugator muscle for the negative stimuli or the zygomaticus muscle for the positive stimuli, and the facial feedback anomaly should predict category-specific deficits in both appraisal and autonomic responses.

## Results

### Explicit emotional ratings (SAM)

A repeated measures ANOVA, with one between-subject factor (group category: HC and PD) and two within-subjects factors (valence: positive vs. negative valence; arousal: high vs. low arousal) was conducted to the subjective rating of valence. This analysis revealed a significant main effect for valence (*F*[1, 55] = 7.09, p ≤ 0.001, ɳ^2^ = 0.38). Each group correctly scored positive stimuli as more positive and negative stimuli as more negative. No other effects were statistically significant (see Table 1).

A repeated measures ANOVA, with one between-subject factor (group category: HC and PD) and two within-subjects factors (valence: positive vs. negative valence; arousal: high vs. low arousal) was applied to the subjective rating of arousal. This analysis showed a significant main effect for arousal (*F*[1, 55] = 11.15, p ≤ 0.001, ɳ^2^ = 0.42). Indeed, there was a clear difference between high vs. low arousing stimuli, as high arousal stimuli were rated as more arousing than low arousal stimuli. No other significant effects were found.

### Skin Conductance Response (SCR)

A three-way repeated measures ANOVA (group x valence x arousal) was applied to compare the SCR values. This analysis showed a significant main effect for valence (*F*[1, 55] = 6.33, p ≤ 0.001, ɳ^2^ = 0.30) and arousal (*F*[1, 55] = 8.15, p ≤ 0.001, ɳ^2^ = 0.37). Negative stimuli induced increased SCR compared to positive stimuli and high arousal stimuli induced higher SCR values than low arousal pictures. Significant interactions between group and valence (*F*[1, 55] = 6.54, p ≤ 0.001, ɳ^2^ = 0.32) and between group and arousal (F[1, 55] = 5.98, p ≤ 0.001, ɳ^2^ = 0.28) were observed. In particular, SCR increased in response to negative more than to positive stimuli in HC (F[1, 33]=4.87, p ≤ 0.001, ɳ^2^ = 0.26). Conversely, in PD, no significant difference was found between positive and negative stimuli (F[1, 21] = 1.02, p = 0.11, ɳ^2^ = 0.10). Moreover, SCR increased for high arousal stimuli as compared to low arousal stimuli for HC (F[1, 33] = 7.77, p ≤ 0.001, ɳ^2^ = 0.31), whereas no differences were found for PD (F[1, 21] = 1.18, p = 0.11, ɳ^2^ = 0.12). In addition, comparing HC and PD, significant differences were found between SCR values in response to negative stimuli (F[1, 55] = 7.70, p ≤ 0.001, ɳ^2^ = 0.32), with greater values for HC than PD. Similarly, HC showed higher SCR values for high arousal stimuli compared to PD (F[1, 55] = 6.47, p ≤ 0.001, ɳ^2^ = 0.28). See Table 1 for details.

### Electromyographic (EMG) measures

#### Zygomatic

**Figure 1:**
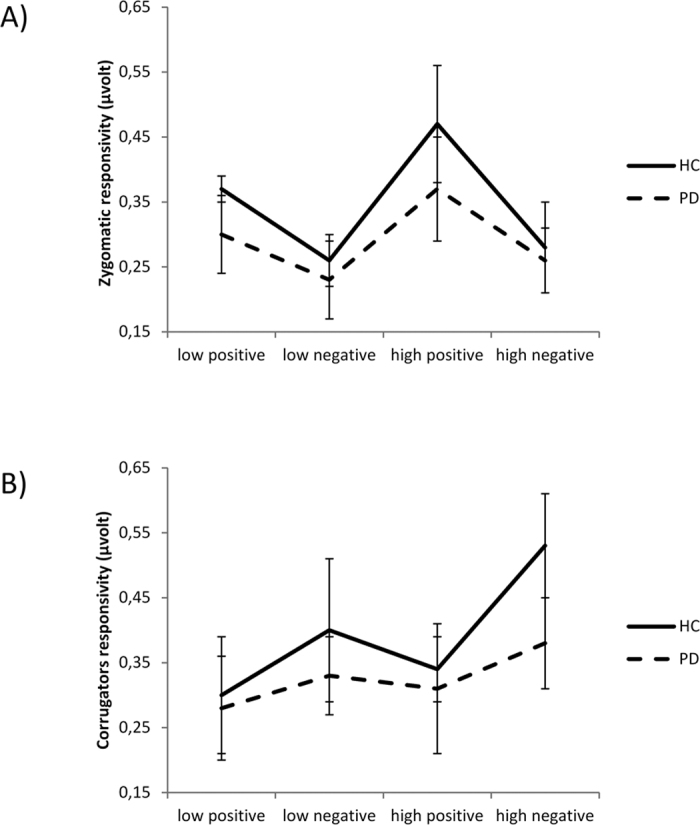
EMG responsivity in Healthy Controls (HC) and Patients with Parkinson’s Disease (PD). A general reduced responsiveness of zygomatic (**A**) and corrugator (**B**) muscles in PD was observed compared to HC. Moreover, we found higher zygomatic activity for positive high arousal stimuli than for positive low arousal and negative stimuli. Higher values of corrugator activity for high arousal negative than low arousal negative and for positive stimuli were recorded. Interestingly, only HC showed increased corrugator activity in response to negative stimuli, while PD patients did not show this reactivity. Finally, HC showed a greater corrugator responsiveness than PD patients for negative stimuli and for high arousal stimuli.

A three-way ANOVA (group x valence x arousal) was applied to zygomatic responsiveness. This analysis showed significant effect for group (*F*[1, 55] = 9.17, p ≤ 0.001, ɳ^2^ = 0.39), with a general reduced zygomatic responsiveness in PD compared to HC. No other effect due to the group factor was significant. Moreover a significant effect for valence was found (*F*[1, 55] = 8.09, p ≤ 0.001, ɳ^2^ = 0.33), with a general greater zygomatic muscle activation in response to positive than to negative stimuli. In addition, a significant interaction between arousal and valence was found (*F*[1, 55] = 10.54, p ≤ 0.001, ɳ^2^ = 0.39), with increased zygomatic activity for positive high arousal pictures as compared to positive low arousal and to negative stimuli (for all comparisons p ≤ 0.001). No other effects were statistically significant. See Fig. 1A for details.

#### Corrugator

A three-way ANOVA (group x valence x arousal) was applied to corrugator responsiveness. This analysis showed a significant effect for group (*F*[1, 33] = 8.02, p ≤ 0.001, ɳ^2^ = 0.35): greater corrugator responsiveness was observed in HC than PD. Moreover, the valence effect was significant (*F*[1, 33] = 6.90, p ≤ 0.001, ɳ^2^ = 0.29), with higher general muscle activity in response to negative stimuli. In addition, the interaction between valence and arousal was significant (*F*[1, 55] = 7.97, p ≤ 0.001, ɳ^2^ = 0.33), with higher corrugator responsiveness for high arousal negative pictures than low arousal negative and positive stimuli (for all comparisons p ≤ 0.001). Interestingly, the interactions between group and valence (*F*[1, 55] = 6.31, p ≤ 0.001, ɳ^2^ = 0.28) and between group and arousal (*F*[1, 55] = 7.03, p ≤ 0.001, ɳ^2^ = 0.32) were significant. Indeed, only HC showed greater corrugator activity in response to negative than positive stimuli (HC: *F*[1, 33] = 7.97, p ≤ 0.001, ɳ^2^ = 0.33; PD: *F*[1, 21] = 1.79, p = 0.11, ɳ^2^ = 0.12). In addition, HC showed a greater corrugator responsiveness than PD for negative stimuli (*F*[1, 55] = 9.05, p ≤ 0.001, ɳ^2^ = 0.37) and for high arousal stimuli (*F*[1, 21] = 8.32, p ≤ 0.001, ɳ^2^ = 0.34). No other effects were statistically significant. See Fig. 1B for details.

### Regression analysis between EMG and SCR measures

Distinct stepwise multiple regression analyses were performed separately for PD and HC. The two predictor variables were corrugator/zygomaticus muscles activity and the predicted variable was SCR modulation in response respectively to high and low arousal positive images, high and low arousal negative emotional cues and neutral stimuli.

**Figure 2:**
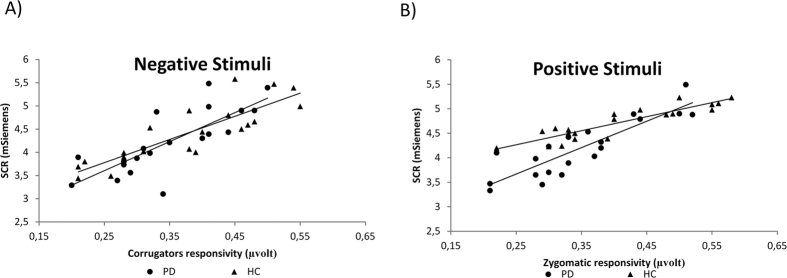
Correlational analyses between SCR and EMG in Healthy Controls (HC) and Patients with Parkinson’s Disease (PD). Significant correlations were found between corrugator/zygomaticus muscles and skin conductance response (SCR). In both HC and PD participants, corrugator activity modulation predicted SCR values in response to negative stimuli (for both high and low arousal) (**A**) and increased zygomaticus response predicted higher SCR values in response to positive stimuli (for both high and low arousal) (**B**).

As shown in Fig. 2, for HC corrugator activity modulation predicted SCR values in response to negative stimuli (high and low arousal), with increased activity for corrugator muscle related to increased SCR. In contrast, for positive stimuli, the correlation analysis was not significant. Conversely, increased zygomaticus response predicted higher SCR values in response to positive stimuli (high and low arousal) but not in relationship with negative stimuli. No significant effects were found for the arousal related measures.

In the patients with PD group, a significant correlation was found between corrugator activity and SCR in response to negative stimuli (high and low arousal): a decreased corrugator activity explained the decreased SCR values. In contrast, no significant affect was found for positive stimuli. Regarding the zygomatic muscle, a significant effect was found exclusively for positive stimuli (high and low arousal), with an increased zygomatic response to positive cues related to increased SCR values, but not for negative cues (Table 2).

### Regression analysis between SCR/EMG measures and valence and arousal

Distinct stepwise multiple regression analyses were performed for PD and HC and for measures of valence and arousal assessed with explicit evaluation of emotions (Self-Assessment Manikin). The predictor variables were corrugator/zygomaticus muscles and SCR activity; the predicted variable were subjective ratings for positive and negative valence for high and low arousal stimuli, respectively

As reported in Table 3, significant increase of corrugator activity and SCR values in response to negative high arousal stimuli and significant increase of zygomaticus activity and SCR values in response to positive high arousal stimuli in HC were found.

### Regression analysis between clinical scales assessing emotional processing and SCR and EMG measures and

Distinct stepwise multiple regression analyses were performed with PD patients clinical data. Predictor variables were Beck Depression Inventory-II (BDI) and Toronto Alexithymia Scale (Tas-20^24^) scores and the predicted variables were SCR modulation and EMG responsiveness in response respectively to high and low arousal positive images, high and low arousal negative emotional cues and neutral stimuli.

The results showed that Tas-20 score predicted SCR values exclusively in response to negative stimuli, with increased dysfunction in emotional awareness related to decreased SCR reactivity (high arousal: R^2^ = −0.53, p = 0.017; low arousal: R^2^ = −0.54, p = 0.015). Furthermore, BDI score predicted SCR values in response to negative stimuli, with increased symptoms of depression related to decreased SCR (high arousal: R^2^ = −0.55, p = 0.011; low arousal: R^2^ = −0.45, p = 0.049). For positive stimuli, no significant correlations were found.

No significant effects were observed for the correlation between BDI and Tas-20 and EMG activity in response respectively to positive and negative, high and low arousal emotional cues.

## Discussion

The present study aimed to elucidate the contribution of central, peripheral and facial response measures in emotional cues processing in PD patients. The integration of several measurements allowed us to a more direct comparison between the explicit appraisal of emotions, with specific reference to the two parameters of valence and arousal, and the autonomic responsiveness to emotional pictures. Moreover, we assessed the role of EMG (zygomaticus and corrugator muscle) in determining the central and peripheral modulation of emotions.

In PD patients, the appraisal processes on both valence (correct positive vs. negative attribution) and arousal (correct dichotomy between high vs. low arousal category) features of emotional cues were preserved, but we found significant decrease in autonomic responses. Specifically, in comparison to healthy subjects, PD patients revealed a reduced responsiveness (lower SCR values) in response to negative and high arousing emotional stimuli. This result was compatible with other previous findings^14^, but partially in contrast with other results which showed a general “blunted effect”^14^,^16^ for both self-reported measures and autonomic behaviour. However, in some studies the EEG measures were found to be unimpaired, pointing out a sort of preservation of central nervous system process and a compromised early automatic process^8^.

Our results suggested a category-specific autonomic impairment: only the negative valence emotional cues induced an anomalous autonomic behaviour although with a preserved explicit appraisal, whereas the positive cues were well processed in terms of both central and peripheral measures. This effect was confirmed by an analogous trend for the arousal category. Indeed, PD patients did not equally respond to high arousal cues as HC, since they “under-estimated” this emotional category by showing reduced SCR responses.

More specifically, PD subjects seem to inadequately respond to the emotional categories which were considered more “salient” (since negative and arousing) for HC subjects. They had preserved appraisal process (explicit rating), but disrupted automatic ability to distinguish between different emotional contexts. This underestimation response related to negative and high arousing emotions was remarked by the autonomic measures without any effect in the explicit measures. These results were further confirmed by the regression analyses. Indeed, it was observed an absence of significant relationship between autonomic measures (EMG and SCR) and valence and arousal ratings in PD, whereas significant effects were found for HC.

This may suggest a partial “detachment” between more central (appraisal) and peripheral (EMG and SCR) processes. This discrepancy may represent a sort of disconnection between two different levels of the emotional processing, namely the spontaneous automatic responsiveness and the controlled appraisal, where the latter appeared well preserved and the former seemed to be impaired.

To explain this effect a third main component was considered in the present research. We evaluated the facial feedback induced by the observation of emotional cues. As revealed by the zygomaticus and corrugator muscle modulations, in line with what was found for SCR, defective response exclusively limited to negative and high arousing emotional category was observed. Indeed, whereas HC increased their corrugator activity in response to negative emotions, patients with PD did not show this behaviour. According to previous evidence, the observation of negative emotional patterns generally induced an increased corrugator muscle response, since this muscle is valence-specific. The absence of an adequate activation of corrugator muscles in PD patients confirmed what we previously found in relation with the autonomic behaviour (i.e. reduced SCR for the negative category).

Therefore, we may state that in PD there is a specific lack of the standard aversion-related behaviour, which in normal conditions is marked by an increased facial corrugator activity and by a prompt increase in autonomic response (SCR) to potentially aversive (negative and high arousing emotions) conditions. In fact, as shown by previous studies, activity of corrugator muscle is generally related to negatively-valenced stimuli, while activity of zygomatic muscle is related to positively-valenced stimuli^18^,^25^,^26^,^27^. In addition, more arousing emotional stimuli generally activate a significant autonomic responsiveness (increased SCR)^28^. This specific “valence effect” and “arousal effects” were absent in PD.

To explain these multiple effects we found, as suggested by “simulation model” of emotions^19^,^25^, when subjects are unable to reproduce the specific behaviour related to the emotional stimulus or context, they also become physiologically “unresponsive” to that context and they end up being emotionally numb. Concurrently, they may maintain the ability to correctly categorize the emotional cues based on their valence/arousal, since this central process appears to be independent from the autonomic system activity. Therefore, the significant absence of a coherent peripheral response (by EMG and SCR modulation) may have impaired this automatic process of simulation of emotions, which generally supports our adequate responsiveness to emotional contexts in healthy subjects. Interestingly, in the regression model, EMG modulation of corrugator/zygomatic muscles affected SCR profile related to a specific valence (zygomatic for positive stimuli; corrugator for negative stimuli). This effect may reinforce the role of the “simulation” of emotions by facial feedback in affecting the arousal variations (as expressed by SCR). In other words, the reduced responsiveness of face (as shown by EMG) may act on the physiological activity which regulates the arousal level (as shown by SCR), reducing the general peripheral responsiveness in PD.

To summarize, in our study, PD subjects seem to inadequately respond to the emotional categories which were considered more “salient” (such as the negative and the arousing ones). They had preserved appraisal process, but disrupted implicit ability to distinguish between different emotional contexts. Therefore, we may suggest a partial independence of the two processes, where the automatic ones may be impaired whereas the central ones may be partially preserved, with significant effects on the behavioural (facial display) level.

This study, that needs further replication, contributes in understanding the underpinnings of emotional reactivity in PD and argues for the impairment of implicit emotional processing. In addition, in our sample of patients with PD we found a predictive negative correlation between clinical scales assessing depression and alexithymia and physiological measures, suggesting that autonomic response decreases together with symptoms of depressions and with inability to identify and describe emotions increase. In particular, depression and alexithymia correlate with SCR selectively during the processing of negative emotional stimuli, which were considered more “salient” for HC subjects. Therefore, we may consider some trait (such as depression and alexithymia) as possible co-factor able to partially affect the HC and PD response, modulating the autonomic reactivity. Future research may better elucidate this relationship.

## Methods

### Participants

Twenty PD patients, fulfilling the UK Parkinson’s Disease Brain Bank criteria for the diagnosis of idiopathic Parkinson’s Disease^29^, were recruited. At the time of recruitment, all patients were being treated with levodopa and/or dopamine agonists. All patients underwent a neuropsychological and clinical evaluation (see Table 4).

Moreover, in all participants the Italian translation of the Italian translation of the Toronto Alexithymia Scale - TAS-20^24^ was carried out, which represents the most widely used measure of the alexithymia construct. The questionnaire consists of 20 self-descriptive statements that must be judged on a 5-point Likert scale ranging from 1 (strongly disagree) to 5 (strongly agree). This scale is divided into three subscales: Difficulty Identifying Feelings, Difficulty Describing Feelings and Externally Oriented Thinking (Table 4).

Patients were always tested in the *on* phase. Stringent exclusion criteria were applied as follows: a) confounding neurological and psychiatric disorders; b) history of traumatic brain injury; c) clinically known hearing or vision impairment or past history of alcohol abuse; d) clinical presentations suggestive of Dementia with Lewy Bodies, Progressive Supranuclear Palsy, Multiple System Atrophy or Vascular Parkinsonism; e) diagnosis of Parkinson Disease Dementia. Patients who scored below 26 out of 30 on the Mini Mental State Examination (MMSE) were also excluded^30^.

In order to assess specific impairments in emotional processing in PD, we also included 34 healthy controls (HC), matched for age and education. All participants were made fully aware about the aims of the research and a written informed consent was sought from all subjects. The work was conducted in accordance with local clinical research regulations and conformed to the Helsinki Declaration. Ethics approval was obtained from the local Ethical Committee (IRCCS Centro San Giovanni di Dio Fatebenefratelli, Brescia, Italy).

### Stimuli

Patients were required to observe and evaluate affective pictures during autonomic parameter recording. Twenty five stimuli were chosen from the International Affective Picture System (IAPS)^31^,^32^, depicting 10 positive (5 high arousal and 5 low arousal), 10 negative (5 high arousal and 5 low arousal) and 5 neutral stimuli, based on valence and arousal ratings obtained from a prior validation study^11^. IAPS subjective ratings were obtained using an adapted 5-point version of the Self-Assessment Manikin (SAM) scale^32^,^33^.

### Procedure

Subjects were seated in a dimly lit room facing a computer monitor and stimuli were presented using Presentation software (Version 14.9, www.neurobs.com).

Participants were required to observe each stimulus during autonomic (SCR) and facial electromyographic (EMG) recordings, and were instructed to attend to the images the entire time they were visible. Three minutes of resting baseline was registered at the beginning of the experiment, before the picture series. Pictures were presented in a random order for 7 seconds, with an inter-stimulus interval of 20 seconds. After stimulus offset, subjects rated their emotional experience on a bipolar SAM scale, evaluating valence and arousal^33^. During an initial familiarization phase, subjects saw and rated five pictures (one for each emotional category), all of which were different from those used in the experimental phase.

### Autonomic measures

A bio-feedback device (Biofeedback 2000, version 7.01) was used to record autonomic activity and facial EMG response. One set of electrodes was connected to the Bio-feedback Amplifier. To measure SCR the skin was cleaned with alcohol and slightly abraded before attaching the 4 mm Ag/AgCl electrodes, which were positioned over the medial phalanges of the second and third finger of the non-dominant hand^34^. SCR elicited by each stimulus was registered continuously with a constant voltage. It was manually scored and defined as the largest increase in conductance during emotional cue presentation, with a cut-off of at least 0.4 μS in amplitude with respect to pre-stimulus mean values. Pre-stimulus values were scored during the 10 seconds prior to stimulus onset.

### Facial electromyographic recording

EMG activity in the zygomatic and corrugator muscles were registered with 4 mm Ag/AgCl electrodes, in accordance with guidelines for psychophysiological recording^35^. The set of electrodes was connected to the Bio-feedback Amplifier with a sampling rate of 1000 Hz. Trials with artefacts were excluded from analysis (2%). Activity of the facial muscles was recorded with a bandpass 20–1000 Hz and rectified and integrated with a contour following integrator. Frequencies of interest generally ranged from 20 to 400 Hz. EMG responses were successively scored as the difference between the mean rectified corrugator/zygomatic signals observed during the presentation of the stimuli and the mean rectified signals prior to stimulus presentation (baseline measure). A positive value indicates that the corrugator/zygomatic measures were greater during the face-viewing phase than during the baseline phase. Trials with artefacts were excluded from analysis.

### Statistical analyses

Statistical analyses were performed using SPSS software (Version 21.0, IBM SPSS Statistics for Windows. Armonk, NY: IBM Corp).

A three-way repeated measures ANOVAs, with one between-subject factor (group category: HC and PD) and two within-subjects factors (stimulus category: high vs. low arousal; and positive vs. negative valence) were applied to the subjective rating of valence and arousal. A three-way ANOVA (group x valence x arousal) was applied to the psychophysiological measures (SCR; EMG). For all of the ANOVAs tests, the degrees of freedom were corrected using Greenhouse-Geisser epsilon when appropriate. Finally, regression analysis was performed between EMG and SCR, between SCR/EMG and subjective ratings of valence and arousal and between SCR/EMG and clinical scales scores (BDI-II and Tas-20).

### Ethics statement

All procedures performed in studies involving human participants were in accordance with the ethical standards of the institutional research committee. Informed consent was obtained from all individual participants included in the study. Ethics approval was obtained from the local Ethical Committee (IRCCS Centro San Giovanni di Dio Fatebenefratelli, Brescsia, Italy).

## Additional Information

**How to cite this article**: Balconi, M. *et al*. Facial feedback and autonomic responsiveness reflect impaired emotional processing in Parkinson's Disease. *Sci. Rep.* **6**, 31453; doi: 10.1038/srep31453 (2016).

## Figures and Tables

**Table 1:** Behavioural and psychophysiological data across groups.

Variable			HC	PD
SAM	Valence(range 1–5)	Positive	4.41 (0.36)	4.44 (0.41)
		Negative	1.81 (0.92)	1.98 (0.74)
	Arousal(range 1–5)	High	3.10 (0.59)	3.32 (0.68)
		Low	1.98 (0.53)	2.01 (0.38)
SCR (mSiemens)	Valence	Positive	5.41 (1.09)	5.18 (1.33)
		Negative	6.13 (1.28)	5.36 (1.22)
	Arousal	High	6.33(1.06)	5.32(0.78)
		Low	4.98 (1.03)	5.23 (0.88)
EMG Zygomatic (μvolt)	Valence	Positive	0.42 (0.05)	0.34 (0.07)
		Negative	0.27 (0.05)	0.25 (0.05)
	Arousal	High	0.38 (0.08)	0.32 (0.07)
		Low	0.32 (0.03)	0.27 (0.06)
EMG Corrugator (μvolt)	Valence	Positive	0.32 (0.05)	0.30 (0.07)
		Negative	0.47 (0.05)	0.36 (0.05)
	Arousal	High	0.44 (0.07)	0.35 (0.08)
		Low	0.35 (0.10)	0.31 (0.07)

Mean values (standard deviations).

HC: Healthy Control; PD: Parkinson’s Disease; SCR: Skin Conductance Response; EMG: electromyography.

**Table 2:** Stepwise multiple regressions.

Predictor	HC		PD	
	*zygomatic*	*corrugator*	*zygomatic*	*corrugator*
Model	1	2	1	2
***negative high arousal***				
R	0.19	0.65	0.30	0.59
R^2^	0.03	0.40	0.09	0.34
β	0.25	0.28	0.13	0.28
std error	0.21	0.12	0.17	0.23
t	1.09	**2**.09*	0.88	**1**.61*
***negative low arousal***				
R	0.28	0.60	0.37	0.62
R^2^	0.07	0.36	0.11	0.37
β	0.32	0.36	0.21	0.24
std error	0.20	0.20	0.18	0.19
t	1.05	**1**.98*	1.02	**1**.54*
***positive high arousal***				
R	0.66	0.72	0.65	0.73
R^2^	0.44	0.50	0.41	0.53
β	0.21	0.22	0.20	0.28
std error	0.18	0.15	0.26	0.30
t	**2**.34*	0.96	**2**.10*	1.09
***positive low arousal***				
R	0.64	0.70	0.58	0.63
R^2^	0.40	0.49	0.34	0.39
β	0.17	0.26	0.22	0.28
std error	0.20	0.21	0.15	0.20
t	**2**.09*	0.89	**1**.98*	1.09

Zygomatic and corrugator as predictor variables, SCR as predicted variable in response to different emotions. *indicates p < 0.05.

**Table 3:** Stepwise multiple regressions.

Predictor	HC			PD		
	*zygomatic*	*corrugator*	*SCR*	*zygomatic*	*corrugator*	SCR
Model	1	2	3	1	2	3
***negative high arousal***						
R	0.20	0.65	0.82	0.03	0.29	0.47
R^2^	0.04	0.40	0.67	0.07	0.09	0.22
β	0.25	0.28	0.30	0.13	0.18	0.20
std error	0.21	0.12	0.11	0.17	0.23	0.20
t	1.09	1.90*	1.44*	0.88	0.90	0.94
***negative low arousal***						
R	0.37	0.46	0.57	0.37	0.51	0.61
R^2^	0.11	0.25	0.33	0.11	0.27	0.38
β	0.22	0.26	0.27	0.21	0.20	0.18
std error	0.20	0.26	0.15	0.18	0.19	0.11
t	1.05	1.03	0.99	1.02	0.95	0.99
***positive high arousal***						
R	0.57	0.66	0.86	0.37	0.48	0.58
R^2^	0.34	0.42	0.71	0.11	0.23	0.31
β	0.18	0.20	0.28	0.20	0.28	0.21
std error	0.18	0.19	0.15	0.26	0.26	0.22
t	1.99*	0.87	1.50*	0.88	1.09	0.94
***positive low arousal***						
R	0.34	0.48	0.61	0.36	0.43	0.52
R^2^	0.13	0.21	0.38	0.10	0.19	0.26
β	0.18	0.22	0.23	0.22	0.21	0.26
std error	0.20	0.22	0.25	0.15	0.20	0.16
t	0.80	0.86	0.94	0.79	1.01	0.80

Zygomatic, corrugator and SCR as predictor variables, subjective rating as predicted variable in response to different emotion. *indicates p < 0.05.

**Table 4:** Demographic characteristics and neuropsychological assessment of patients with Parkinson’s Disease (N = 20).

*Demographic and clinical features*			*Cut-off
Age (years)		68.4 (7.3)	
Gender (male/female)		12/8	
Education (years)		8.7 (3.3)	
Duration of disease (years)		7.6 (3.7)	
Unified Parkinson Disease Rating Scale (UPDRS – III)		30.2 (12.1)	
Hoehn and Yahr Stage		2.3 (0.5)	
***Cumulative Illness Rating Scale** (**CIRS***)			
CIRS, Severity		1.6 (0.2)	
CIRS, Comorbidity		3.4 (1.5)	
Parkinson’s Disease Quality of Life Questionnaire-39		24.0 (14.6)	
Beck Depression Inventory-II		11 (6.5)	<14
Toronto Alexithymia Scale (Tas-20)		48.4 (11.9)	<50
***Neuropsychological Assessment***	***Raw score***	***Adjusted score***	*Cut-off
***Screening for dementia***			
Mini Mental State Examination (MMSE)	27.7 (2.0)	26.7 (2.2)	≥24
***Parkinson’s Disease Cognitive Rating Scale** (**PD-CRS***)			
PD-CRS, Total Score	73.5 (19.5)		>81
PD-CRS, Cortical Score	24.8 (4.4)		
PD-CRS, Subcortical Score	48.6 (15.8)		
***Memory***			
Paired Associates Learning (PAL) - *Cantab*	86.6 (34.1)		
Story Recall	9.0 (3.3)	11.0 (3.2)	>7.5
Digit Span	5.3 (1.0)	5.4 (0.9)	>3.5
***Language***			
Fluency, phonemic	31.5 (12.8)	35.4 (11.4)	>16
Fluency, semantic	33.6 (11.3)	38.4 (9.9)	>24
***Attentional and Executive Functions***			
Trail Making Test, A	75.4 (62.8)	54.5 (63.0)	<94
Trail Making Test, B	245.3 (156.1)	175.2 (148.1)	<283
Frontal Assessment Battery	14.9 (3.2)	15.5 (3.2)	>13.4

^*^Cut-off scores according to Italian normative data are reported. Values are mean ± SD. Bold data indicate scores below cut-off.
